# Crossregulation and Functional Redundancy between the Splicing Regulator PTB and Its Paralogs nPTB and ROD1

**DOI:** 10.1016/j.molcel.2007.06.016

**Published:** 2007-08-03

**Authors:** Rachel Spellman, Miriam Llorian, Christopher W.J. Smith

**Affiliations:** 1 Department of Biochemistry, University of Cambridge, 80 Tennis Court Road, Cambridge CB2 1GA, UK

**Keywords:** RNA

## Abstract

Among the targets of the repressive splicing regulator, polypyrimidine tract binding protein (PTB) is its own pre-mRNA, where PTB-induced exon 11 skipping produces an RNA substrate for nonsense-mediated decay (NMD). To identify additional PTB-regulated alternative splicing events, we used quantitative proteomic analysis of HeLa cells after knockdown of PTB. Apart from loss of PTB, the only change was upregulation of the neuronally restricted nPTB, resulting from decreased skipping of nPTB exon 10, a splicing event that leads to NMD of nPTB mRNA. Compared with knockdown of PTB alone, simultaneous knockdown of PTB and nPTB led to larger changes in alternative splicing of known and newly identified PTB-regulated splicing events. Strikingly, the hematopoietic PTB paralog ROD1 also switched from a nonproductive splicing pathway upon PTB/nPTB knockdown. Our data indicate crossregulation between PTB and its paralogs via nonproductive alternative splicing and a large degree of functional overlap between PTB and nPTB.

## Introduction

Alternative splicing (AS) allows multiple mRNA isoforms to be produced by individual genes. These commonly encode distinct protein isoforms, thereby enriching the repertoire of proteins available to organisms (Black, 2000; Lareau et al., 2004; Maniatis and Tasic, 2002; Matlin et al., 2005). AS can also quantitatively control gene expression by producing mRNAs that are degraded by nonsense-mediated decay (NMD) (Green et al., 2003; Hillman et al., 2004), a phenomenon that appears to be widespread among splicing regulators themselves (Lareau et al., 2007; Ni et al., 2007) and which we refer to here as AS-NMD.

Alternative splicing decisions are influenced by enhancers and silencer *cis*-acting elements that mediate their activity by binding activator and repressor proteins. Activators are commonly members of the SR family of proteins (Graveley, 2000), whereas hnRNP proteins often act as repressors. One of the better-investigated mammalian repressor proteins is polypyrimidine tract binding protein (PTB, also known as PTBP1 and hnRNP-I, reviewed in Wagner and Garcia-Blanco, 2001). PTB represses many exons, including a number of muscle and neuron-specific exons (e.g., Ashiya and Grabowski, 1997; Chan and Black, 1997; Perez et al., 1997; Wollerton et al., 2001). PTB binds to splicing silencers with core sequences consisting of UCUU, CUCUCU, or related motifs (Chan and Black, 1997; Perez et al., 1997). In some cases, a single silencer is sufficient to mediate PTB repression (Izquierdo et al., 2005; Shen et al., 2004), but most PTB-regulated exons have multiple PTB binding sites. Most mammals contain two PTB paralogs that are expressed in a tissue-restricted manner. nPTB/brPTB/PTBP2 is expressed mainly in neurons (Kikuchi et al., 2000; Markovtsov et al., 2000; Polydorides et al., 2000), whereas ROD1 is expressed in hematopoietic cells (Yamamoto et al., 1999). Rodents contain an additional paralog, smPTB (Gooding et al., 2003). PTB and its paralogs share >70% amino acid sequence identity and a common arrangement of four RRM-type domains. The activity of nPTB has been most extensively studied in the context of the neuron-specific splicing of the N1 exon of *SRC* where it is less repressive than PTB (Markovtsov et al., 2000). Nonetheless, it is likely that nPTB and the other paralogs act as repressors of at least some exons (Polydorides et al., 2000).

PTB itself is subject to alternative splicing. Inclusion or skipping of exon 9 produces the PTB1 and PTB4 isoforms, which can vary in activity (Robinson and Smith, 2006; Wollerton et al., 2001). In contrast, exon 11 skipping produces a frameshifted mRNA that is degraded by NMD (Wollerton et al., 2004). PTB protein itself promotes PTB exon 11 skipping in an autoregulatory feedback loop. The equivalent nPTB exon is also skipped (Rahman et al., 2002; Tateiwa et al., 2001) and has a similar arrangement of potential regulatory elements, suggesting that it could also be regulated by PTB and/or nPTB (Gooding et al., 2006; Wollerton et al., 2004). Indeed nPTB exon 10 lies within one of the genomic ultraconserved elements that have been associated with AS-NMD events (Lareau et al., 2007; Ni et al., 2007).

A range of global approaches have recently been harnessed with the aim of deciphering “cellular codes” composed of particular complements of splicing regulators and “RNA codes” composed of particular arrangements of regulatory sequence modules that together define cell-specific programs of splicing (reviewed in Blencowe, 2006; Matlin et al., 2005). A typical approach involves identification of the set of RNAs bound by a regulatory factor (e.g., Ule et al., 2003) or the set of splicing events affected by the factor. The latter can be identified by perturbing the cellular levels of the splicing regulator and then analyzing RNA with splice-sensitive microarrays (Blanchette et al., 2005; Johnson et al., 2003; Pan et al., 2004; Relogio et al., 2005; Sugnet et al., 2006; Ule et al., 2005). Such analysis has been carried out for *Drosophila* SR and hnRNP proteins (Blanchette et al., 2005) and the mammalian neuron-specific Nova proteins (Ule et al., 2005). As a complementary approach, we decided to use quantitative gel-based proteomics to analyze the consequences of PTB knockdown. Ideally, alterations in the levels of alternatively spliced isoforms would appear as pairs of reciprocally varying spots on 2D gels. Alterations in individual spots would be consistent with AS-NMD events (Wollerton et al., 2004) or with PTB's documented roles at other levels of gene expression, including 3′ end processing (Castelo-Branco et al., 2004; Moreira et al., 1998), regulation of translation (Mitchell et al., 2005), and RNA stability (Hamilton et al., 2003; Pautz et al., 2006).

Despite the weight of evidence for the widespread roles of PTB, we observed very little effect of PTB RNAi upon the HeLa cell proteome. The single exception was upregulation of the usually neuronal nPTB, which resulted from a large increase in nPTB exon 10 inclusion. In model systems of PTB-regulated splicing, double knockdown of both PTB and the upregulated nPTB caused greater changes in AS than knockdown of PTB alone. Strikingly, we found that inclusion of cassette exon 2 of ROD1, which is essential for generation of an mRNA with an open reading frame initiating at the first AUG codon, was also upregulated by knockdown of PTB and nPTB together. Moreover, proteomic analysis showed numerous alterations in protein expression upon PTB+nPTB double-knockdown cells and allowed us to identify novel PTB/nPTB-regulated events. Our data indicate that nonproductive AS is used in both autoregulation and crossregulation of PTB, nPTB, and ROD1 and that nPTB is able to replace many of the functions of PTB in HeLa cells.

## Results

### RNAi against PTB Results in an Increase in nPTB Protein Levels

**Figure 1:**
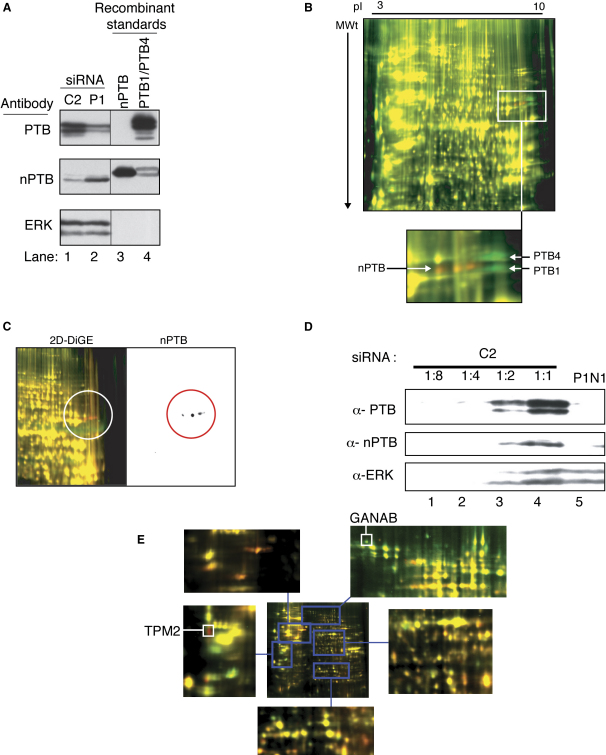
nPTB Protein Is Upregulated by PTB Knockdown (A) Western blot probed for PTB (top panel), nPTB (middle panel), or ERK (bottom panel). Lanes 1 and 2 contain an equivalent amount of protein after RNAi with control C2 siRNA (lane 1) or with P1 siRNA against PTB (lane 2). Recombinant His-tagged nPTB (10 ng, lane 3) or PTB1 and PTB4 (10 ng, lane 4) were loaded as antibody specificity controls. (B) Merged 2D DiGE image of C2- and P1-treated samples. C2 sample was Cy3 labeled, and P1 Cy5 labeled (red). Proteins were separated in the pI range 3–10, nonlinear. Spots that consistently changed upon dye swap, technical repeat, and with a second PTB siRNA are highlighted in the inset. (C) High MWt/pI corner of 2D DiGE image of control and PTB knockdown samples (left panel) showing increasing spots (red) and western blot of same gel probed for nPTB (right panel). (D) Western blot probed for PTB (top panel), nPTB (middle panel), or ERK (bottom panel). Lanes 1, 2, and 3 contain 1:8, 1:4, and 1:2 dilutions of the control sample for estimation of knockdown efficiency. N1 is an nPTB-specific siRNA. Lanes 4–6 contain an equivalent amount of protein. (E) 2D DiGE of proteins from control (Cy3 labeled, green) and PTB+nPTB knockdown (Cy5 labeled, red) cells. The whole gel is shown in the center, with enlarged sections around the periphery. The identity of two sequenced spots is indicated.

Our initial aim was to carry out a proteomic analysis of HeLa cells in which PTB had been knocked down. RNAi was performed using a PTB-specific short-interfering RNA (siRNA) duplex, P1, and a control duplex, C2 (Wollerton et al., 2004), resulting in ∼80%–90% PTB knockdown (Figure 1A, top panel, and data not shown). Samples were analyzed by 2D-difference gel electrophoresis (DiGE) (Tonge et al., 2001; Unlu et al., 1997). The control protein sample was labeled with Cy3 fluorescent dye, and the PTB-RNAi sample with Cy5. The two samples were then pooled and separated on a 2D gel over a pI range of 3–10 (Figure 1B), and two fluorescent images were taken. After false coloring of the merged images, proteins whose levels have changed between the two samples appear red or green, whereas yellow spots indicate proteins whose levels have remained constant. Despite our expectation that knockdown of PTB would cause numerous changes in the HeLa proteome, very few up- or downregulated proteins were observed. The only consistent changes that were observed with reciprocal dye labeling, biological and technical repeats, and with a second PTB siRNA (P2) were a group of spots in the high pI (>9) and 55–60 kDa molecular weight range (Figure 1B and Figure S1 in the Supplemental Data available with this article online). The downregulated spots (green in Figure 1B) corresponded in size and pI to the PTB1 and PTB4 isoforms. We suspected that the adjacent upregulated spots might correspond to one of the PTB paralogs. This might account for the lack of other observed changes if the paralog were able to compensate for the loss of PTB. Comparison of the predicted MWt and pI of nPTB and ROD1 suggested that the upregulated spots were likely to correspond to nPTB. Western blot analysis of control and PTB-RNAi-treated samples confirmed that PTB knockdown was accompanied by an increase in nPTB protein levels (Figure 1A, lane 2, middle panel). Western blot of the 2D gel with nPTB-specific antibodies confirmed that the upregulated spots were nPTB (Figure 1C). The run of spots of the same size but varying pI is likely to correspond to phosphorylation variants of nPTB. Analysis of PTB and nPTB levels after treatment with varying doses of P1 siRNA indicated an inverse relationship between PTB and nPTB levels, with nPTB progressively increasing as PTB decreased (Figure S2). In order to test whether the upregulated nPTB compensates for loss of PTB, we carried out double knockdown of both PTB and nPTB (Figure 1D). 2D DiGE comparing control and PTB+nPTB knockdown cells showed numerous up- and downregulated spots (Figure 1E). By contrast with the limited proteomic consequences of PTB knockdown (compare Figures 1B and 1E), this suggests that the upregulated nPTB buffers against the effects of loss of PTB.

### PTB Regulates nPTB Levels by Alternative Splicing Linked to NMD

Skipping of exon 10 of nPTB produces an mRNA that is a predicted NMD substrate. nPTB exon 10 has a similar arrangement of splice site elements to PTB exon 11 with a predicted distant branchpoint 389 nt upstream of exon 10 (Gooding et al., 2006) and multiple surrounding potential PTB binding sites (Figure S3). We therefore analyzed nPTB exon 10 splicing as a candidate cause of the nPTB upregulation in PTB knockdown cells.

**Figure 2:**
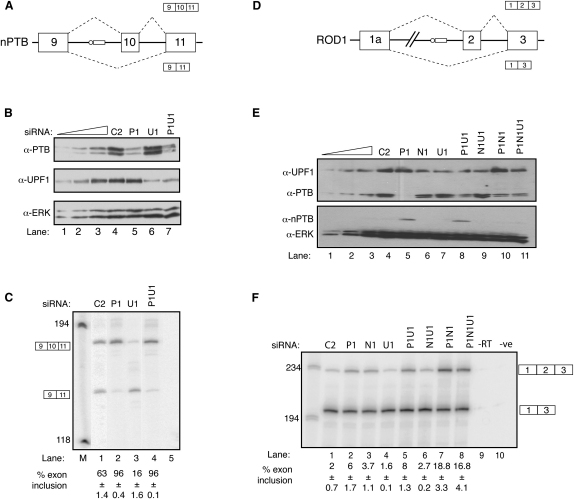
Regulation of PTB Paralogs by Nonproductive Alternative Splicing (A) Schematic representation of alternative splicing of exon 10 of nPTB. Exon skipping leads to introduction of a premature stop codon. Note that, in all cartoon depictions in the manuscript, splicing pathways promoted by PTB are shown at the bottom. (B) Western blot probed for PTB (top panel), UPF1 (middle panel), or ERK (bottom panel, loading control). Lanes 4–7 contain an equivalent amount of protein after treatment with the following siRNAs: C2 siRNA (lane 4), PTB (lane 5), UPF1 (lane 6), and PTB+UPF1 (lane 7). Lanes 3, 2, and 1 contain progressive 2-fold dilutions of the C2 control sample. (C) RT-PCR analysis of nPTB alternative splicing on RNA samples harvested from HeLa cells after RNAi with control C2 siRNA (lane 1) or with siRNAs against PTB (lane 2), UPF1 (lane 3), or PTB+UPF1 (lane 4). Lane 5, PCR negative control. Values below lanes indicate percentage of exon 10 inclusion. Numbers to the left are size standards in lane M. The two amplicons are 182 and 148 nt. All error bars are mean ± SD, n = 3. (D) Schematic representation of ROD1 exon 2 alternative splicing. (E) Western blot probed for UPF1 and PTB (top panel) and nPTB and ERK (bottom panel) from samples treated with siRNAs as in (F). Lanes 4–11 contain an equivalent amount of protein from each particular sample. Lanes 1–3 contain serial 2-fold dilutions of the control sample. (F) RT-PCR analysis of ROD1 exon 2 alternative splicing on RNA samples harvested from HeLa cells after RNAi with the same siRNAs as in (E). All error bars are mean ± SD, n = 3.

RT-PCR analysis revealed a substantial increase in the percentage of nPTB exon 10 inclusion from 63% to 96% upon PTB knockdown (Figure 2C, lanes 1 and 2), consistent with repression by PTB of nPTB exon 10. Similar results were obtained with a second PTB siRNA (data not shown). Skipping of nPTB exon 10 generates a predicted NMD substrate, so RT-PCR analysis likely underestimates exon 10 skipping. We therefore performed RNAi of UPF1, an essential NMD factor (Maquat, 2004), reducing its levels by 80%–90% (Figure 2B). This resulted in a decrease in the observed levels of nPTB exon 10 inclusion to 16% (Figure 2C, lanes 1 and 3), confirming that nPTB exon 10 skipping leads to NMD. A second UPF1 siRNA showed similar results (data not shown). Comparison of UPF1 knockdown alone with UPF1/PTB double knockdown (Figure 2C, lanes 3 and 4) illustrates the full extent of the effect of PTB on alternative splicing of exon 10 of nPTB—an increase in exon inclusion from 16% to 96%. Quantitative real-time PCR analysis revealed that the absolute levels of nPTB RNA were elevated 4.4-fold upon PTB knockdown, in reasonable agreement with the increases in nPTB protein revealed by 2D DiGE (2.4- ± 0.6-fold, Figure S1) and by western blot (∼3-fold, Figure 1A). Taken together, these data demonstrate that, under the influence of repression by PTB, nPTB in HeLa cells is predominantly spliced to skip exon 10, resulting in NMD of the majority of its RNA.

We also asked whether splicing of the hematopoietic-restricted PTB paralog ROD1 might be regulated by PTB and nPTB. Unlike PTB exon 11 and nPTB exon 10, ROD1 exon 10 shows no EST evidence of alternative splicing and appears to have a conventionally located branchpoint 21 nt upstream with little sequence conservation further upstream. In contrast, ROD1 exon 2 has a number of features in common with the regulated PTB and nPTB exons. It is identical in length (34 nt), is skipped in many ESTs, and has a conserved region of nearly 200 nt upstream, which encompasses an AG dinucleotide exclusion zone of 168 nt (Gooding et al., 2006), candidate distant branchpoints up to −160, and multiple potential PTB binding motifs (Figure S3). Indeed, we found exon 2 to be mainly skipped in HeLa cells (Figure 2F). Its inclusion was slightly upregulated upon knockdown of PTB or nPTB alone and was included to the greatest extent upon knockdown of PTB and nPTB together (Figure 2F). Inclusion of ROD1 exon 2 produces an mRNA with a single open reading frame initiating in exon 1, whereas exon 2 skipping leads to introduction of a premature stop codon in exon 3. UPF1 knockdown had no effect upon the ratio of exon 2 inclusion or skipping, suggesting that the exon 2-skipped isoform is not an NMD substrate. This could be because the stop codon is only 20 codons from the initiator AUG (Silva et al., 2006). Nevertheless, the exon 2-skipped isoform is unlikely to be efficiently translated as the short open reading frame would repress translational initiation further downstream. Therefore, not only is nPTB expression regulated by PTB-induced AS-NMD in HeLa cells, but ROD1 is regulated by both PTB and nPTB.

### nPTB Compensates for PTB in Alternative Splicing of PTB, α-Actinin, and α-Tropomyosin Pre-mRNA

**Figure 3:**
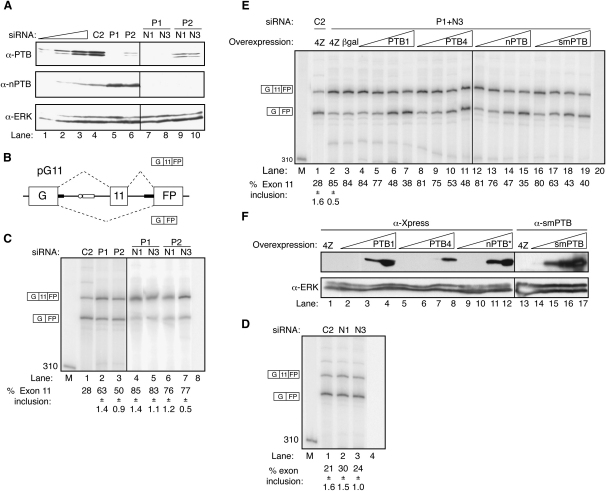
PTB/nPTB Knockdown Increases PTB Exon 11 Inclusion (A) Western blot probed for PTB (top panel), nPTB (middle panel), or ERK (bottom panel). Lanes 4–10 contain an equivalent amount of protein after RNAi with negative control C2 siRNA (lane 4) or with siRNAs against PTB (P1, P2, lanes 5 and 6) or PTB (P1, P2) and nPTB (N1, N3) (lanes 7–10). Lanes 3, 2, and 1 contain progressive 2-fold dilutions of the C2 control sample. (B) Schematic representation of pG11 PTB minigene reporter with PTB exon 11 flanked by partial wild-type intronic sequences (thin black lines) and GFP exons. PTB promotes the skipped pathway (Wollerton et al., 2004). (C) RT-PCR analysis of pG11 alternative splicing in HeLa cells after RNAi with control C2 siRNA (lane 1) or siRNAs against PTB (lanes 2–3) or PTB+nPTB (lanes 4–7). Lane 8, PCR negative control. Numbers to the left are size standards in lane M. The two amplicons are 406 and 372 nt. All error bars are mean ± SD, n = 3. (D) RT-PCR analysis of pG11 alternative splicing in HeLa cells after RNAi with negative control C2 siRNA (lane 1) or with siRNAs against nPTB (N1, lane 2; N3, lane 3). Lane 4, PCR negative control. All error bars are mean ± SD, n = 3. (E) RT-PCR analysis of pG11 alternative splicing in HeLa cells after RNAi with negative control C2 siRNA (lane 1) or with P1+N3 siRNAs against PTB and nPTB (lanes 2–20). Increasing amounts (1, 10 100, and 800 ng) of expression plasmid for PTB1 (lanes 4–7), PTB4 (lanes 8–11), nPTB (lanes 12–15), or smPTB (lanes 16–19) were cotransfected. pGEM4Z (lanes 1 and 2) and pCMV-β-gal (lane 3) were negative controls. Lane 20, PCR negative control. Triplicate repeats were only carried out for lanes 1 and 2. All error bars are mean ± SD, n = 3. (F) Western blot analysis of protein harvested from HeLa cells treated with C2 control siRNA and overexpression of increasing amounts (1, 10, 100, and 800 ng) of expression plasmid for PTB1 (lanes 2–5), PTB4 (lanes 6–9), nPTB (lanes 10–13), or smPTB (lanes 15–18). Lanes 1–12 were probed with anti-Xpress antibody, and lanes 14–18 were probed with anti-smPTB antibody.

The 2D DiGE data and the analysis of ROD1 exon 2 splicing suggested some functional redundancy between PTB and nPTB. To examine this further, we investigated the effects of PTB and PTB+nPTB knockdown upon three characterized systems of PTB-regulated splicing. Splicing of PTB exon 11 was analyzed by using an NMD-insensitive minigene reporter (pG11; Figure 3B) (Wollerton et al., 2004). Knockdown of PTB resulted in an increase in exon 11 inclusion (Figure 3C, compare lanes 2 and 3 to 1). When the increase in nPTB protein levels was prevented by double knockdown of PTB and nPTB, there was an even larger increase in exon 11 inclusion (Figure 3C, lanes 4–7), suggesting that nPTB represses PTB exon 11. As expected, treatment with nPTB siRNAs (N1 and N3) alone had little effect on splicing (Figure 3D).

We next tested the ability of transfected PTB and nPTB to reverse the effects of PTB+nPTB knockdown (Figures 3E and 3F). P1 siRNA targets the PTB 3′UTR, which is not in the expression constructs, whereas nPTB was expressed from a codon-optimized construct (F. Robinson and C.W.J.S., unpublished data), which was resistant to the nPTB siRNAs. Add back of PTB1, PTB4, nPTB, and smPTB all reversed the effects of double knockdown (Figure 3E, lanes 12–15). This demonstrates that nPTB is equally repressive as PTB for repression of PTB exon 11.

**Figure 4:**
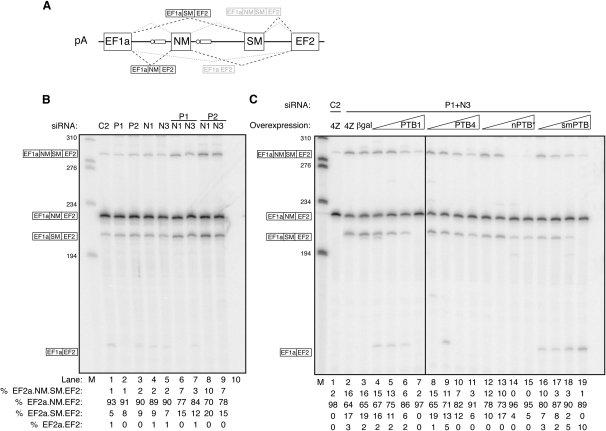
PTB/nPTB Knockdown Switches Splicing of α-Actinin (A) Schematic representation of pA α-actinin minigene reporter containing α-actinin EF1a, NM, SM, and EF2 exons and complete introns (thin black lines). PTB represses SM exon inclusion. (B) RT-PCR analysis of pA alternative splicing in HeLa cells after RNAi with negative control C2 siRNA (lane 1) or with siRNAs against PTB (lanes 2 and 3, P1 and P2), nPTB (lanes 4 and 5, N1 and N3), or PTB+nPTB (lanes 6–9). Lane 10, PCR negative control. Numbers to the left indicate size markers (nt) in lane M. The four amplicons shown schematically to the left are 289, 223, 208, and 142 nt in size. The percentage of each the four products is shown below each lane. For ease of presentation, values are rounded to whole numbers and SD are not shown. (C) RT-PCR analysis of pA alternative splicing in HeLa cells after RNAi with control C2 siRNA (lane 1) or with siRNAs against PTB and nPTB (lanes 2–19). Increasing amounts (1, 10, 100, and 800 ng) of expression plasmids for PTB1 (lanes 4–7), PTB4 (lanes 8–11), nPTB (lanes 12–15), or smPTB (lanes 16–20) were cotransfected. pGEM4Z (lanes 1 and 2) and pCMV-β-gal (lane 3) were negative controls. Lane 20, PCR negative control. Only lanes 1–3 were carried out as triplicate repeats. Note that lane 2 in (C) is equivalent to lane 7 in (B). The lower effects of siRNAs P1 and N3 in (B) were associated with a lower degree of nPTB knockdown than in (C) (data not shown).

Splicing of α-actinin was analyzed with the pA minigene, which contains the NM and SM alternative exons flanked by the constitutive EF1a and EF2 exons (Figure 4A) (Southby et al., 1999). RNAi of PTB had no detectable effects upon pA splicing in many experiments and only minor effects in others (Figure 4B, lanes 2 and 3). Treatment with nPTB siRNAs was also without effect (Figure 4B, lanes 4 and 5). When the increase in nPTB was prevented by double knockdown, there was a substantial switch in splicing, with an increase in double NM and SM inclusion, a decrease in NM inclusion, and an increase in SM inclusion (Figure 4B, lanes 6–9). Overexpressed PTB1, PTB4, nPTB, and smPTB were all able to reverse the effects of knockdown (Figure 4C, lanes 7, 11, 15, and 19).

**Figure 5:**
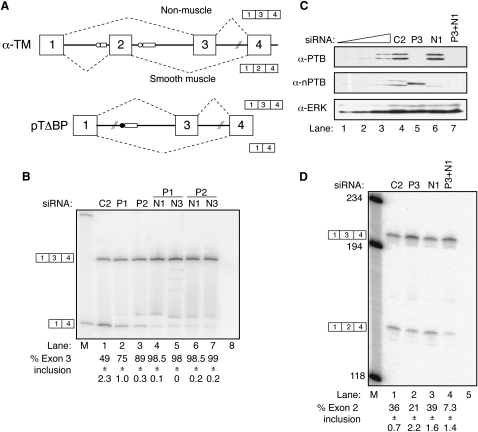
PTB/nPTB Knockdown Decreases α-TM Exon 3 Skipping (A) Schematic representation of α-TM mutually exclusive splicing (top) and pTΔBP construct (below). Exon 3 is included by default in nonmuscle cells. Exon 2 is included in smooth muscle due to repression of exon 3 by PTB. The black circle indicates the point mutations in the exon 3 branchpoint that allow detection of exon skipping in HeLa cells. Crosshatched lines indicate sequences deleted in the construct, including exon 2. (B) RT-PCR analysis of pTΔBP alternative splicing in HeLa cells after RNAi with control C2 siRNA (lane 1) or with siRNAs against PTB (lanes 2 and 3, P1 and P2) or PTB+nPTB (lanes 4–7). Lane 8, PCR negative control. Lane M, size markers (603 and 310 nt shown). Amplicons shown schematically to the left are 435 and 309 nt. All error bars are mean ± SD, n = 3. (C) Western blot probed for PTB (top panel), nPTB (middle panel), or ERK (bottom panel). Lanes 4–7 contain an equivalent amount of protein after RNAi in rat PAC1 cells with negative control C2 siRNA (lane 4) or with rat-specific siRNAs against PTB (lane 5), nPTB (lane 6), or PTB+nPTB (lane 7). Lanes 3, 2, and 1 contain progressive 2-fold dilutions of the C2 control sample to allow the degree of knockdown to be estimated. (D) RT-PCR analysis of endogenous α-TM alternative splicing in RNA samples harvested from rat PAC1 cells after RNAi with negative control C2 siRNA (lane 1) or with siRNAs against PTB (lane 2), nPTB (lane 3), or PTB+nPTB (lane 4). Lane 5, PCR negative control. Samples were digested with XhoI after PCR. 1-3-4 product 194 nt, 1-2-4 product 145 nt. Numbers to the left indicate the size markers in lane M. All error bars are mean ± SD, n = 3.

At the 5′ end of the αTM gene (HUGO *TPM1*), exon 2 is specifically included in smooth muscle due to PTB-mediated repression of exon 3 (Gooding et al., 1998; Wollerton et al., 2001). HeLa cells do not transcribe *TPM1*, and transfected constructs do not skip exon 3. However, the pTΔBP reporter construct with point mutations in the exon 3 branchpoint produces substantial amounts of exon 3-skipped product (Gooding et al., 2006), which is dependent upon the characterized negative regulatory elements (C. Gooding and C.W.J.S., unpublished data). Whereas knockdown of PTB alone reduced the levels of exon skipping (Figure 5B, lanes 1–3), PTB+nPTB knockdown reduced skipping to less than 2% (lanes 4–7). The effects of PTB knockdown upon endogenous *Tpm1* splicing were analyzed in rat PAC1 smooth muscle cells (Rothman et al., 1992). Knockdown of PTB was again accompanied by an increase in nPTB (Figure 5C, middle panel, lane 5), confirming that the crossregulation of PTB and nPTB is not species or cell specific. Rat nPTB was knocked down with the N1 siRNA (Figure 5C, lane 7). Knockdown of PTB alone resulted in a decrease in TM exon 2 inclusion (Figure 5D, lane 2), whereas N1 siRNA had no effect. Double knockdown of PTB+nPTB once again produced the largest effect with an overall 5-fold decrease in exon 2 inclusion (Figure 5D, lane 4).

### Proteomic Identification of PTB and nPTB Targets

**Figure 6:**
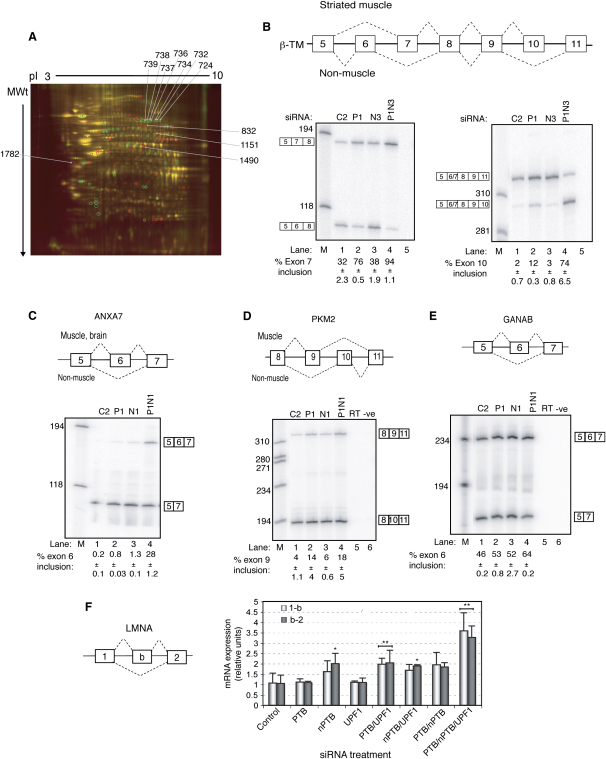
Proteomic Identification of PTB Targets (A) Representative 2D gel from the Biological Variance Analysis. The gel was loaded with 100 μg of protein from each of the Cy2 internal standard, Cy3 control, and Cy5 PTB+nPTB knockdown. Up- and downregulated spots, identified by analysis of the six gels, are indicated by red and green circles, respectively. Numbered spots correspond to proteins resulting from alterations in alternative splicing. Note that the gel is imaged in all three channels, so consistently up or downregulated spots may not appear red or green. (B–E) RT-PCR validation of PTB/nPTB-regulated alternative splicing events in the TPM2, ANXA7, PKM2, and GANAB genes. In each case, a schematic cartoon of the alternative splicing event is shown at the top. RNA samples were harvested from HeLa cells after RNAi with control (C2, lane1), PTB (P1, lane 2), nPTB (N1, lane 3), or PTB+nPTB (P1N1, lane 4) siRNAs. Numbers to the left are size markers in lane M. All error bars are mean ± SD, n = 3. (F) Schematic representation of *LMNA* alternative splicing in the region between exons 1 and 2 (left). Q-RT-PCR analysis of RNAs from HeLa cells treated with siRNAs against PTB, nPTB, UPF1, or combinations. The data presented are means of primer pair expression normalized relative to HPRT expression ± SD of three biological replicates. Light gray bars represent primer pair between exons 1 and b, dark gray represents primer pair between exons b and 2. Asterisk indicates significant differences from control, t test p < 0.05.

The preceding data show that knockdown of both PTB and nPTB is necessary to maximize the chances of identifying PTB targets. Although 2D DiGE was useful for preliminary characterization of proteomic changes upon PTB+nPTB knockdown (Figure 1E), intergel variation (e.g., Figure S1) can make reliable identification of up- or downregulated spots difficult. We therefore adopted a more sophisticated procedure, biological variance analysis (BVA) (Alban et al., 2003; Karp et al., 2005), which incorporates a Cy2-labeled internal pooled standard comprising equal aliquots of all control and knockdown samples. Six gels were run with the pooled standard and the Cy3- and Cy5-labeled biological replicate samples (Figure S4) (Alban et al., 2003). To reduce the likelihood of identifying secondary events, protein was harvested 48 hr earlier than in the preceding experiments. We identified 81 regulated spots (p < 0.05), of which 16 were upregulated and 15 downregulated more than 1.2-fold (Figure 6A and Figure S4). Note that, in contrast to the DiGE gels (Figure 1), the gel in Figure 6A is imaged with all three channels, so spots that are confidently identified as up- or downregulated do not appear red or green. A number of these spots were picked for LC-MS/MS sequencing. In some cases, the identified peptides clearly indicated the presence of specific protein isoforms. For example, spot 1782 was upregulated 1.6-fold and contained multiple β-tropomyosin (TPM2) peptides, including some diagnostic of muscle-specific exons 7 and 10. TPM2 had also been identified from the initial DiGE gels (Figure 1E). RT-PCR analysis verified that splicing of the two mutually exclusive exon pairs 6 and 7, and 10 and 11 was altered substantially upon PTB+nPTB knockdown, with an intermediate response to PTB knockdown (Figure 6B). In both cases, splicing was switched toward the muscle-specific exons 7 and 10, consistent with the peptide data. Spot 1490 contained annexin 7 (ANXA7) peptides and was downregulated 1.3-fold. Exon 6 of ANXA7 is a cassette exon used primarily in striated muscle and brain. The position of spot 1490 is consistent with the 50 kDa exon 6-skipped isoform. Peptides diagnostic of exon 6 inclusion or skipping were not identified. Nevertheless, RT-PCR showed a substantial shift toward exon 6 inclusion in double knockdown, but not in single PTB or nPTB knockdown (Figure 6C), again consistent with the proteomic data. Spot 1151 was downregulated 1.3-fold and contained peptides corresponding to two isoforms of pyruvate kinase 3 (PKM2) containing mutually exclusive exon 9 or 10. RT-PCR demonstrated ∼4-fold increased inclusion of PKM2 exon 9 relative to exon 10 in response to either PTB or PTB+nPTB knockdown (Figure 6D). Because the two isoforms comigrate and are present in the same spot, in this case it is not clear how a shift in the ratio of mutually exclusive exons could explain the observed downregulation of spot 1151. α-glucosidase II (GANAB) was identified in a downregulated (∼1.3-fold) spot from the original 2D DiGE gels (Figure 1E) and in spot 485 from the BVA experiment. GANAB cassette exon 6 was found to be partially upregulated by either PTB or nPTB knockdown and to show greater upregulation in the combined knockdown (Figure 6E). Although no isoform-specific peptides were identified, the position of the downregulated protein spot was consistent with the exon-skipped isoform, again indicating agreement between the protein and RNA data. Finally, a charge train of downregulated spots (724, 734, 736–9, −1.2- to −1.5-fold) all contained peptides corresponding to lamin A (*LMNA*). Extensive alternative splicing at the 3′ end gives rise to the major lamin A/C isoforms. However, an alternative 5′ end exon (chr 1 154,363,150–154,363,330) usually associated with an internal promoter appeared to be a better candidate PTB-regulated exon. Splicing of this exon between the usual exons 1 and 2 would lead to introduction of a premature stop codon, leading to NMD. RT-qPCR indicated that splicing of exon b to both exon 1 and exon 2 (Figure 6F, light bars and dark bars, respectively) increased by 2-fold with PTB+nPTB knockdown and by 4-fold with PTB+nPTB+UPF1 knockdown. Inclusion of this exon could account for the downregulation of the LMNA/C-containing spots. One additional spot (832) containing LMNA/C was upregulated. This could be due to other proteins present in the same spot.

Therefore, quantitative proteomic analysis has allowed identification of a number of PTB-regulated alternative splicing events, most of which are regulated by both PTB and nPTB, but with varying sensitivities to single or combined knockdown.

## Discussion

The data presented here shed interesting light upon crossregulation between PTB, nPTB, and ROD1, as well as indicating a high degree of functional redundancy between PTB and nPTB. PTB was already known to autoregulate its own expression by repressing its own exon 11 (Wollerton et al., 2004). The feedback loop may serve to prevent overexpression of PTB, to upregulate expression upon PTB export to the cytoplasm, and to reduce stochastic noise in PTB expression (Becskei and Serrano, 2000). Although splice-sensitive microarray analysis of cells knocked down for UPF1 has been used to argue against the widespread harnessing of regulated alternative splicing to NMD (Pan et al., 2006), more recent computational and array analyses have indicated that AS-NMD is widespread among both SR proteins and hnRNP proteins, where it is associated with ultraconserved genomic regions (Lareau et al., 2007; Ni et al., 2007). Similar findings had previously been reported with plant SR protein genes (Kalyna et al., 2006). These reports speculate that the AS-NMD events might generally be involved in autoregulatory feedback loops, as previously demonstrated for PTB (Wollerton et al., 2004) and SC35 (Sureau et al., 2001). Our findings demonstrate that such nonproductive splicing events can also be harnessed to crossregulate the expression of families of regulators. In HeLa cells, PTB switches nPTB off by promoting skipping of nPTB exon 10 (Figures 2A–2C), while PTB and nPTB both promote the nonproductive splicing of ROD1 (Figure 2F). Although nPTB exon 10 is associated with an ultraconserved genomic region, PTB exon 11 and ROD1 exon 2 also have extended conserved upstream regions that coincide with extended AG exclusion zones, indicative of branchpoint sequences remote from the exon (Gooding et al., 2006) (Figure S3). The reason for coincidence of ultraconserved regions with AS-NMD events is a mystery. However, for the PTB, nPTB, and ROD1 exons, the extended regions of upstream conservation can be explained in part by the distant branchpoint arrangement and the likely location of regulatory elements.

nPTB protein is usually expressed with neuronal specificity in the retina and brain (Kikuchi et al., 2000; Markovtsov et al., 2000; Polydorides et al., 2000; Rahman et al., 2002), although nPTB transcripts can be detected in cells in which nPTB protein is not present (Boutz et al., 2007a). The regulated skipping of nPTB, PTB, and ROD1 exons provides a mechanism for the cell to respond to excessive levels of any or all of the PTB paralog proteins. The relative sensitivities of the exon-skipping events appear to be tuned to facilitate PTB expression in preference to nPTB or ROD1. Thus the normal levels of PTB protein in HeLa cells result in 80% inclusion of PTB exon 11 (Wollerton et al., 2004), 16% inclusion of nPTB exon 10, and 2% inclusion of ROD1 exon 2 (Figure 2). Reduced PTB protein levels may therefore be a prerequisite for nPTB or ROD1 protein expression. In our experiments, this was achieved by PTB knockdown in HeLa and PAC-1 cells, but in vivo, this might result from developmentally programmed reduction in PTB transcription (Boutz et al., 2007b). We also showed that nPTB protein is equally effective as a repressor of PTB exon 11 (Figure 3) and so potentially could modulate PTB levels. Whether this control is actually exerted in vivo is an open question. The crossregulation and partial functional redundancy of PTB and its paralogs will have implications for the analysis of transgenic knockout mice. On the one hand, many of the normal functions of the knocked-out factor may be masked by compensatory upregulation of the paralog(s). However, this may have the advantage of restricting phenotypes to the events that are critically dependent upon the individual paralog that has been knocked out.

Expression of nPTB appears to be intricately regulated at multiple levels, in addition to the PTB-induced exon 10 skipping. For example, upregulation of the miR133 family of miRNAs during striated muscle differentiation translationally silences nPTB expression (Boutz et al., 2007a). The reduction in PTB+nPTB activity thereby allows various muscle-specific splicing events to occur, such as *Tpm2* exon 7 and 10 selection (Figure 6B). Another unusual feature of nPTB expression, shared by ROD1, is that it has an extremely suboptimal codon content, with a high proportion of codons ending in A or U, which are relatively infrequent in the human genome (Lander et al., 2001). This limits its ability to be translated in vitro or overexpressed in transfected cells and may also modulate its expression in vivo. Indeed we were only able to overexpress nPTB (Figures 3 and 4) by using a codon-optimized construct with 211 silent mutations (F. Robinson and C.W.J.S., unpublished data).

The fact that very few changes were observed in 2D DiGE analysis of PTB knockdown samples unless nPTB was also knocked down (Figure 1) suggests a high extent of functional redundancy between PTB and nPTB in HeLa cells. The functional redundancy is not surprising in view of the >70% amino acid identity between PTB and its paralogs. Nevertheless, it contrasts with the example of the *SRC* N1 exon, where nPTB is much less repressive (Markovtsov et al., 2000). PTB and nPTB also have a differential effect upon internal ribosome entry segment (IRES) driven translation (Guest et al., 2004; Mitchell et al., 2003; Pilipenko et al., 2001). It may be that N1 is representative of a subset of exons that are differentially sensitive to PTB nPTB, whereas many other splicing events are equally repressed by either paralog. Among the AS events that we analyzed (Figures 2–6), the sensitivity to knockdown of PTB alone varied, with substantial effects upon PTB exon 11 (Figure 3) but negligible effects upon actinin splicing (Figure 4). It may be that there is a continuum of AS events with differential sensitivities to repression by PTB or nPTB. Presumably, this differential sensitivity to PTB and nPTB is written into an “RNA code” and might be decipherable upon analysis of a sufficient number of splicing events that are coregulated (Blencowe, 2006; Matlin et al., 2005; Ule et al., 2006). A similar situation probably pertains with events regulated by PTB and ROD1 in immune cells (Lynch, 2004).

The role of PTB in regulating splicing of FGFR2 exon IIIb (Wagner and Garcia-Blanco, 2002) and PTB exon 11 (Wollerton et al., 2004) has been demonstrated previously by RNAi of PTB. However, before uncovering the upregulation of nPTB upon PTB knockdown, we were unable to obtain convincing RNAi evidence for the role of PTB in regulating *Tpm1* (Figure 5) or α-actinin (Figure 4) splicing. Likewise the previous evidence for PTB regulation of mammalian *TPM2* exon 7 was based upon PTB binding to an intronic silencer rather than functional evidence. Splicing of a chicken β-TM construct in HeLa cells was shown to respond partially to RNAi of PTB (Sauliere et al., 2006). The lack of complete derepression was taken to indicate the involvement of other repressor factors. Our data suggest that the additional repressor is nPTB.

Our data suggest that quantitative proteomics might provide a useful complementary approach to methods for global analyses of alternative splicing (Blencowe, 2006; Matlin et al., 2005). Here we identified eight splicing events that are targets of PTB in the *PTBP2*, *ROD1*, *TPM2*, *ANXA7*, *LMNA*, *PKM2*, and *GANAB* genes (Figures 2,6). The *TPM2* and *PKM2*-regulated exons are usually muscle specific, whereas *ANXA7* exon 6 is used in muscle and brain. This is consistent with previous indications that PTB commonly represses muscle and brain-specific exons. It will be interesting to see whether a strong enrichment of muscle and neuron-specific exons is maintained as we increase the number of validated PTB-regulated events. Likewise, although the number of validated events currently prevents a robust analysis for enrichment of particular regulatory sequence motifs, it is clear that the newly discovered PTB-regulated events share some common features with the previously characterized examples (Figure S3). These include a high frequency of known PTB binding motifs and potential branchpoint sequences located beyond the conventional ∼40 nt distance from the exon. We aim to compare analysis at the proteome level with the use of splice-sensitive microarrays (Blanchette et al., 2005; Johnson et al., 2003; Pan et al., 2006; Relogio et al., 2005; Sugnet et al., 2006; Ule et al., 2005) in order to rigorously determine the extent to which proteomic analysis can contribute to understanding the global roles of splicing regulators. In view of the fact that PTB and other splicing regulators also influence gene expression at other levels, including translation (Mitchell et al., 2005; Sanford et al., 2004), it seems likely that proteomic analysis will usefully complement splice-sensitive transcriptome analysis. Nonetheless, the data presented here already demonstrate that unanticipated insights can be gained from this approach.

## Experimental Procedures

### Cell Culture and Transfection

Minigene reporter constructs pG11, pA, and pTΔBP have been described previously (Gooding et al., 2006; Southby et al., 1999; Wollerton et al., 2004). Constructs for overexpression of PTB1, PTB4, nPTB, and smPTB were generated from pCMV-β-gal by replacing the β-gal ORF with human PTB1, PTB4, nPTB, or rat smPTB. The PTB1 and PTB4 constructs also contained the His_6_ and XP tags from the pcDNA3.1/HisC vector. Human nPTB was overexpressed by using a codon-optimized construct (F. Robinson and C.W.J.S, unpublished data).

HeLa and PAC1 smooth muscle cells (Rothman et al., 1992) were cultured under standard conditions in DME medium with glutamax and 10% fetal calf serum. siRNA transfection of HeLa cells was carried out as described (Wollerton et al., 2001) with the following siRNAs: 10 pmol P1, N1, N3, and U1 and 110 pmol P2, all made up to total 120 pmol with C2. siRNA transfection of PAC1 cells was the same, except 3 × 10^4^ cells were seeded, siRNA transfection used 2 μl oligofectamine (Invitrogen), and cells were harvested on day 6. mRNA targets for gene specific knockdown were as follows: human PTB P1 and P2 (Wollerton et al., 2004) and rat PTB P3, 5′-GCCUCUUUAUUCUCUUCGG-3′; human nPTB N1, 5′-GAGAGGAUCUGACGAACUA-3′; human nPTB N3, 5′-UAAGAAACCUGGAUCCAAA-3′; human UPF1 U1 (Mendell et al., 2002) and human UPF1 U2, 5′- GCUCCUACCUGGUGCAGUAUU-3′ control C2 (Wagner and Garcia-Blanco, 2002). siRNAs were purchased from Dharmacon.

### Protein Harvest and Analysis

Protein was harvested with RIPA buffer supplemented with protease inhibitors. Protein concentration was determined by the Bradford method. Proteins were separated on 15% SDS-PAGE gels, transferred to PVDF membrane, and detected by enhanced chemiluminescence. Primary antibodies used were mouse anti-PTB BB7 monoclonal for HeLa cells, rabbit anti-PTB polyclonal for PAC1 cells, rabbit anti-nPTB, rabbit anti-smPTB, mouse anti-Xpress (Invitrogen), rabbit anti-UPF1, and rabbit anti-ERK (Santa Cruz Biotechnology Inc) with Donkey anti-rabbit and anti-mouse IgG horseradish peroxidase conjugate secondary antibodies (Jackson ImmunoResearch Laboratories Inc). Where necessary, western blot images were scanned with a flat-bed scanner, and band intensities were quantitated with Phoretix 1D Advanced software.

### RNA Harvest and Analysis

RNA was harvested with TRI reagent (Sigma) (Wollerton et al., 2001), and splicing patterns were analyzed by RT-PCR. RNA was treated with DNase I (Ambion) before reverse transcription using either 200 U Superscript II (Invitrogen) or 10 U AMV RT (Promega) (Southby et al., 1999; Wollerton et al., 2001). Oligonucleotides for RT were CGRT, 5′-TAGTTGTACTCCAGCTT-3′, for pG11, and SVRT, 5′-GCAAACTCAGCCACAGGT-3′, for pA, oligo dT, or random hexamers for endogenous mRNAs. PCR was carried out on 1/25 of the RT reaction. Standard PCR conditions were 30 cycles 94°C 30 s, annealing 1 min, 72°C 1 min. Other conditions are given in Table 1. In all cases, the reverse primer was ^32^P end labeled (except for the first round PCR of pA and PKM2), and labeled products were resolved on 5% polyacrylamide gels (12% for Figures 6C and 6D) and quantitated by Molecular Dynamics Storm 840 phosphorimager using Molecular Dynamics ImageQuant software. Quantitative PCR (QPCR) was performed on a Rotor-GeneTM 6000 (Corbett Research) with SYBR green (Applied Biosystems) after reverse transcription of 1 μg total RNA using Superscript II (Invitrogen) and oligo dT. Fold change in mRNA levels was calculated relative to the control sample and normalized to GAPDH levels. QPCR of endogenous nPTB transcripts was performed as a two-step reaction. Except where otherwise indicated, values for exon inclusion are shown as mean ± SD of three biological repeats.

### 2D DiGE and Biological Variance Analysis

Protein samples were harvested in ASB14 lysis buffer (8 M urea, 2% ASB14, protease inhibitors 1 mM PMSF, 1 mg/ml aprotinin, and 1 mg/ml leupeptin). CyDye labeling, 2D protein separation, gel imaging, and analysis were performed as described previously (Karp et al., 2004). Gels were stained with colloidal Coomassie, and proteins were excised manually. In the BVA experimental set up (Figure 6 and Figure S4), six biological replicates from control or PTB+nPTB siRNA-treated cells were analyzed. CyDye labeling, 2D gels, and analysis were performed as described in Karp et al. (2005). Gels were stained with Sypro Ruby, and proteins were excised with an automated spot picker. Proteins were digested with trypsin before analysis by LC-MS/MS sequencing (Cambridge Centre for Proteomics, Department of Biochemistry, University of Cambridge). MS/MS fragmentation data were used to search the NCBI primary sequence database using Mascot search engine (Perkins et al., 1999).

## Figures and Tables

**Table 1:** PCR Primers and Conditions

Primer Name	Sequence (5′ → 3′)	Reporter/GOI	Annealing Temperature (°C)	Product Sizes (bp)	MgCl2 (mM)
CG5′	GGCAAAGAATTCGCCACCA	pG11	58	372 and 406	1.5
CG3′	GGGTGTCGCCCTCGAACTT				
SV5′	GGAGGCCTAGGCTTTTGCAAAAAG	pA	62		2.5
SV3′	ACTCACTGCGTTCCAGGCAATGCT	pTΔBP			
ACT5′	ATCAGCCAGGAACAGATG	pA nested	60	142, 208, 223, 289	1.5
ACT3′	ACATGAAGTCAATGAAGGCYTG				
TM1	CGAGCAGAGCAGGCGGAG	Tpm1	60	145,194 (XhoI digest)	1
TM4	CAGAGATGCTACGTCAGCTTCAGC				
nPTB5′	GAGTGGGTATGCCTGGAGTCT	nPTB	62	148, 192	3
nPTB3′	GTTTCCATCAGCCATCTGTATTA				
BTM5	GAAGCTGGTGATCCTGGAAGGA	TPM2	60	109,185 (PvuII digest)	2
BTM8	CCAACAGTTTGATCTCCTCTT				
BTM10	CTTGTACTTCATCTTCTGGGCAT	TPM2	60	304, 330	2
BTM11	GTCTGGTCCAAGGTCTGGTGAA				
GANAB_ex5 s	GGATTAACCATGGCTGAGG	GANAB	58	171, 237	2
GANAB_ex7as	TCAGCTGGGTCTTTTGATCC				
PKM2_ex9 s	GCATCATGCTGTCTGGAGAA	PKM2	58	141, 338 (PstI digest)	2
PKM2_ex12as	GAAGATGCCACGGTACAGGT				
ANXA7_ex5 s	CTTTCCTGGAGGACAGATGC	ANXA7	58	105, 170	1.5
ANXA7_ex7as	GCTGGTCGGATAGTTCCTTG				
ROD1_ex1as	TGTTCCCACTCTCCTTCCAC	ROD1	58	201, 234	1.5
ROD1_ex3as	GGTCCGTTAATGATGCCAGA				

QPCR		Validated Event			

LMNA_ex1	CCGAGTCTGAAGAGGTGGTC	LMNA Junction exons 1 and b			
LMNA_exb	TCTTCCCATCACTGGCAAAT				
LMNA_exbs	AGGATGCAAGGGAAAGGACT	LMNA Junction exons b and 2			
LMNA_ex2	AGGTCACCCTCCTTCTTGGT				
nPTBQ5′	TCTGCCACCCTTCACCTATC	nPTB (PTBp2) gene expression			
nPTBQ3	TCTTCCACTGTTGCCATCTG				

## Note Added in Proof

Similar findings of crossregulation between PTB and nPTB have recently been reported by Black and colleagues (Boutz et al., 2007b).

## Supplemental Data

Document S1. Four Figures
