# Supplementary material for: Crossregulation and Functional Redundancy between the Splicing Regulator PTB and Its Paralogs nPTB and ROD1
Source: Mol Cell. 2007 Aug 3;27(3):420–34. doi: 10.1016/j.molcel.2007.06.016 (PMC1940037; doi:10.1016/j.molcel.2007.06.016)
Supplement: Document S1. Four Figures [file mmc1.pdf]

Molecular Cell, Volume *27*

Supplemental Data

Crossregulation and Functional Redundancy between the Splicing Regulator PTB and Its Paralogs nPTB and ROD1

Rachel Spellman, Miriam Llorian, and Christopher W.J. Smith

Figure S1. Reproducibility of nPTB upregulation upon PTB knockdown.

The region of 2D DiGE gels containing PTB and nPTB. Left and right columns represent reciprocal labeling experiments. The top left panel is the same as in Fig 1. Row 2 is a technical repeat of Row 1 (i.e. same protein samples run on two more gels). Row 3 are nuclear extract fractions from a biological repeat. Row 4 gels were from samples generated by use of a second PTB siRNA (P2). Note that nPTB is reproducibly upregulated in all samples. PTB was resolved on all gels, probably due to its high pI (>9.7) which is close to the upper range of the pI strips used.

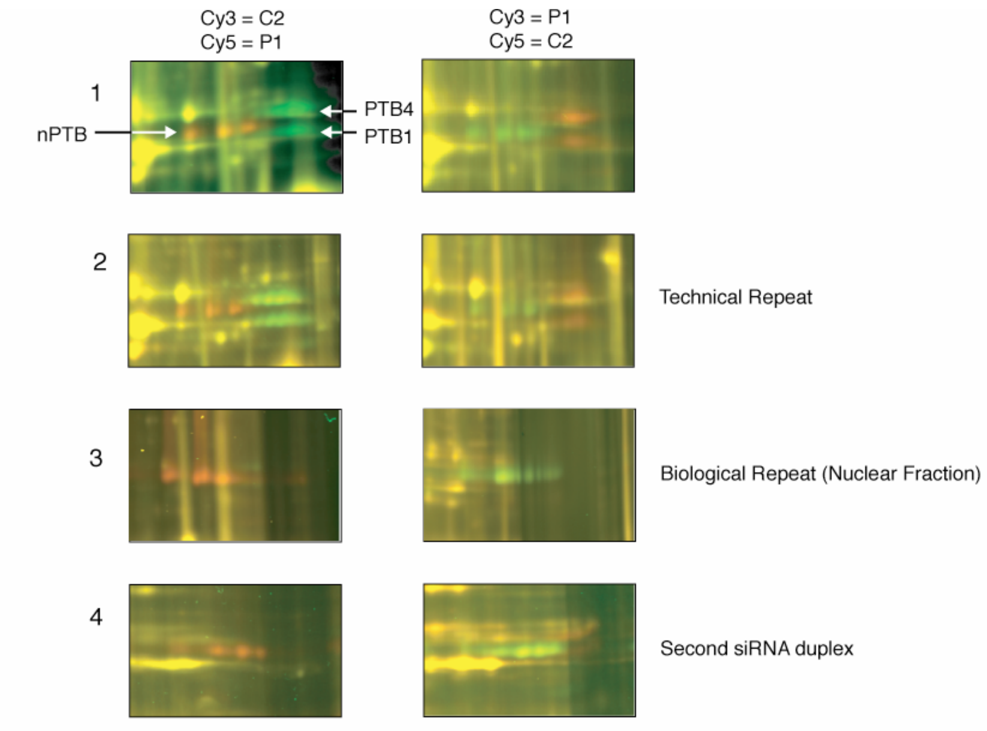

Figure S2. Relationship between PTB knockdown and nPTB upregulation.

A) RNAi against PTB was carried out with the indicated amounts of P1 siRNA (lanes 5-10). Lane 4 is an equivalent amount of protein from HeLa cells treated with the C2 control siRNA. Lanes 3, 2, and 1 are 2-fold dilutions of the C2 sample to allow estimates of knockdown.

B) Graphical representation of % nPTB increase *vs*. % PTB knockdown.

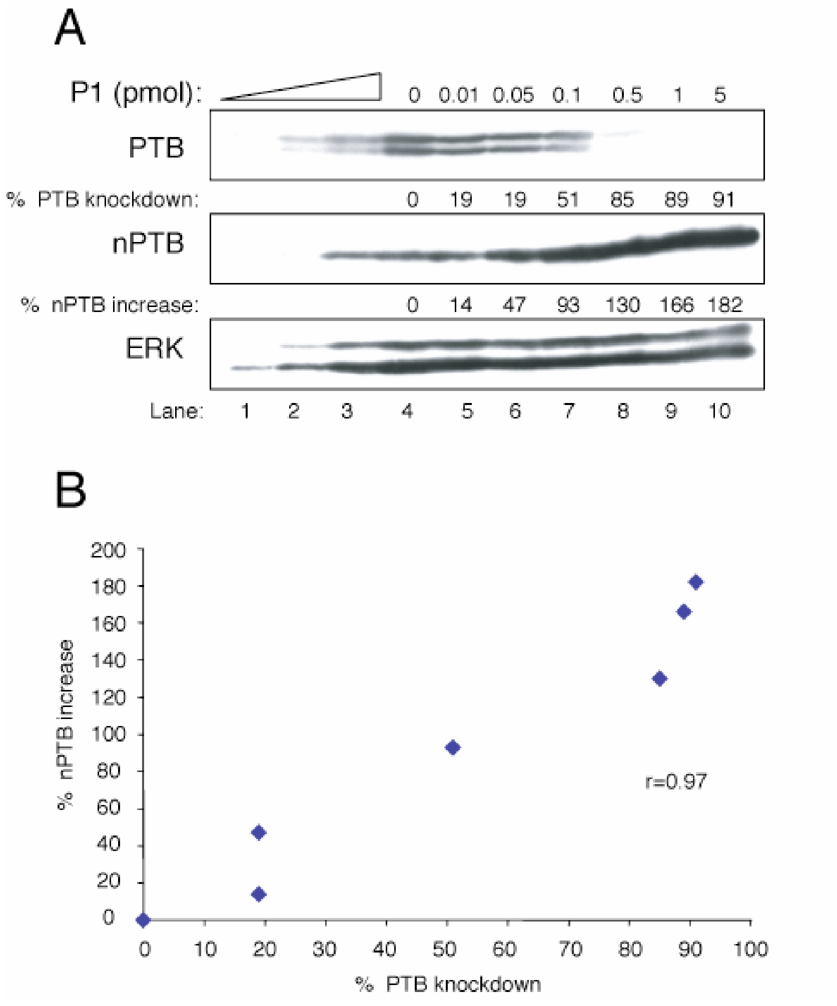

Figure S3. Sequences associated with PTB-regulated exon. Sequences are shown of all PTB/nPTB regulated exons shown in this manuscript along with associated intron sequences. Exon sequences are shown in upper case, introns in lower case. In many cases, the sequence shown extends to the third upstream AG dinucleotide, with AGs shown in blue. Defining an AG exclusion zone in this way helps to focus on cases where the branch point may be far upstream (Gooding et al., 2006). Branch point sequences (verified or predicted) are indicated in pink, while potential PTB binding motifs (TCTT, CTCTCT or TCTCT) are shown in red, except in some cases where such a motif overlaps branch point sequences (ROD1 exon 2, TPM2 exon 10, PKM2 exon 9). In LMNA exon "b" the red TAG is a stop codon when exon b is spliced to exon 1, while the green ATG would be the start codon when LMNA transcripts initiate from an internal promoter. Regions shaded gray show very high levels of conservation indicated by the UCSC genome browser 17 vertebrate species conservation track.

PTB (PTBp1) Exon 11

gaggtaaacctgtaactggaatgtgtgtggagtgtgactgatagaacactacctgattcttatgtattt
actgacctgtgtttttttgctactttttttcttttctccccttcccctttccctattttttttcttgcc
ctgatccggaatttctttgccaactgactgcacggtacttctgcttcctgttgttgcttgaaacaaaac
aaaaacataaacaaataaaaaacaaaaattccccctcaaaccctgctctccggaaaccaacctgccctt
gaatattaacatcctgacaacttcatcatccatcaaccactgcacgcctgcggggactgtcttcctcgt
gtggacgattggcaactcgcccccccttgacctctccctctcccctgtccctccgctgccttgctctgct
gtctctaaag
AGAGTCACACCCCAAAGCCTCTTTATTCTTTTCG

nPTB (PTBp2) Exon 10

tagatgtactttacccatatttgtcttggatgactacattttactcagttttttcttgtacagtacataa
accaaccattttctgaccaaattcctgcatttcctatgtactgacctatatttattttgtttttgttc
cccaattccttattttttttcttctgcattgctgtttcccttccccatttcatcctttttccctgtgtgtt
caccttccctttccttgtcttttcccaaatgcccattcccttccctgtcttatcctttattttccttgt
ccttgtcttcattccctgtctccattccctatgttcatgcttctgtgcttgaacaaatgttcctcggac
caacttgccccaattaaccgccttgaaccatgatccatgaccacctcaccattctgcgggaaccaccct
tcgttatggatgatctgttcatctccgctcttcctcgactcttctctcttcttgtcttacgctgcttgc
tcttctctccttctaaag
ATGGTTACGCCCCAAAGTCTGTTTACCCTCTTCG

ROD1 Exon 2

tagtaaggatttgcttttgcactctgctgttgctagactgtctcatcattttctttgtattaatattt
cacaactcatcacattctttattttattatttgttattataatgcttttttcccctttattctttactatc
tctgtcttttaaaaacttgttctctaactttgttttccatttttctctttttacttttttccatacag
TGTTGTTACAGATCTTATAACAGTCGGTTTAAAG

Actn1 (Rat) SM Exon

gagcacgctcaagggtggcctctggggcgagaaataactcatttctcctttttttcttctctcttctgt
ctgaattctccctgtctcccttcctgtgggccatggggctcatcctctcctgctctccctgcctgcccc
ctgtcgggttcctctgacttctgacctctgacttcggctatcttttcctgggaacctgcttgggtatccg
cctcctctccctcgtcacatctcacctgtggactggtcttctgcatttctttgctctgacctccttcaa
catgttcctcctacattcttcggctcctcctaatcttcacaccataaaccgggcccttcctctttctgc
tgtcctcctggcctctgcctgggccctcctcctcccacctgtctgtccctcctgtgtcttggcaccact
gcccacag
AAGAAGACGGGCATGATGGACACGGATGATTTCCGCGCCTGCCTTATCTCCATGGGTTACAACATG

Tpm1 (Rat) Exon 3

gtacccgggcgcgcggtgtggcactgcacacgaatggctaactttctctttctctctccctccctgtct
ttccctctctctctctttcccgctgtccctgtcctttatggtctacgcaccctcaacccgcaccttgcg
ggatcacgctgcctgctgcaccccacccccttccccctccttccccccaccccccgtactccactgcca
actccccag
CTGGAAGATGAGCTGGTGTCACTGCAAAAGAAACTCAAGGGCACTGAAGATGAACTGGACAAATACTCC
GAGGCTCTCAAAGATGCCCAGGAGAAACTGGAGCTGGCGGAGAAAAAGGCCACAGAT
gtaagtgcacgctcacactgcctccctcacccccctgaccgcgtggccgctctggggggtcaccacagggg
ctgcagagcaaaggaagagggtgatcctcctcctacaggacacctgcacacagcctggccatagcccag
agcactggatgccgcctctgctgctgcgcacatttcatttatattctgtcctttcccctttttctcctc
ttctttacctcctcccccttt

TPM2 Exon 7

caggaagggcctggggcttctgagccctgcctgctgtcactctcacaactttgctcttctcttctctcc
tccacccgtcccctgctgtgccatctccactgtctggcttcgctgtgccctcacaccctgccccccccg
ccacacacccccctgcag
TAAATGTGGGGACCTAGAGGAGGAGCTGAAAATTGTTACCAACAACTTGAAATCCCTGGAGGCCCAGGC
GGACAAG

TPM2 Exon 10

gagttcctgacagctgtccagcctgttccctggcctccctctctcactctgtctttgtgtacctctgat
tctgttttttctttccattgctctctctccttcctttcctctttccctccactccctttcccacactccc
acctggtcccctccctggaccccccccag
ATGAAGTCTATGCCCAGAAGATGAAGTACAAGGCCATTAGCGAGGAACTGGACAACGCACTCAATGACA
TCACCTCCCTCTGAGCCCCACGCCAGCGTGGCCACCTCAGCTCTCTTCTCTCCTCTCCTTTCCATTCTC
TCTATGGGGA

ANXA7 Exon 6

tgaggtttccatgggtcggaggaaatggcccattatagtaatggaagactaaagtctttacttctgata
gactccatactttctttggtccatctgctttaatagtgtcctgatgactttgagaagacaagcataggt
ctattctcataggtaatgattctcacaactttatcctggagattaacctggggtcttggttttagttct
ttttcctcttccttgcccttaccagcagttacacctatgaatttgggtggctgggtgattggtatttct
gacagtgatttatgtagggtggttttgtttcccattttaccttttataacttttcactcttttttcag
ATCAATACAGATTCTTTTTCTTCCTATCCTGTTTTCTCTCCTGTTTCTTTGGATTATAGCAGTGAA

PKM2 Exon 9

cagatttagccaattaacctaaggttaccttcctctctgataaattccccattctgtcttcccatgtgt
tgtgtctcgtttttttcctcctccttccctcttccttgcccccctcttcccctaaaccttacag
ATAGCTCGTGAGGCTGAGGCAGCCATGTTCCACCGCAAGCTGTTTGAAGAACTTGTGCGAGCCTCAAGT
CACTCCACAGACCTCATGGAAGCCATGGCCATGGGCAGCGTGGAGGCTTCTTATAAGTGTTTAGCAGCA
GCTTTGATAGTTCTGACGGAGTCTGGCAG

GANAB Exon 6

gaggcctgcctgggtctgtgtctcctttcttccccttctcacatcatcacatttcccagatttattttct
tcgttttatttatttattttttttggtttcttttccccccatctctggaagtttgtcgactgaaact
cacttttatgtttctgtttttgtgttggttttgtgcctcttttccccttccctacccatctcctcctg
ccccag
TTTCTCGGATAAGGTTAATCTCACGCTTGGTAGCATATGGGATAAGATCAAGAACCTTTTCTCTAG
gtaaatccatggccaccggtactgtatctgttcctctgcccttaccccattcctcact

LMNA Exon “b”

cagctcttcagacccctgccttgggtcacatttgcaagtgccaactctcatttctaccttattctttttc
ctctctgttcccctccccacccccctctcttccctctttctgagatcag
ATTTGCCAGTGATGGGAAGAGTTAGAAACAGGATGCCCAGCCCTTCTCGCCTCAAGAGGCCACTGGGAT
GCAGCCACTCCTGTGCTTGGGGAACCTGGAGGATGCAAGGGAAAGGACTGGCACTCTGCTGGCACAGCA
CCCGGCCTGGGGCAGGACACGGGCGAAGCCAGGGTCTCCCCT

Figure S4. BVA analysis workflow of PTB+nPTB treated cells.

Workflow for Biological Variance Analysis (BVA) of PTB+nPTB siRNA treated cells. Six biological replicate experiments were carried out. A pooled internal standard was created using equal aliquots of all control and knockdown samples. The pooled standard was labeled with Cy2 and the control and knockdown with Cy3 or Cy5. Six 2D-PAGE gels were run, each with a mixture of Cy2, Cy3 and Cy5 labeled samples. The abundance of individual spots was measured as their Cy3/Cy2 or Cy5/Cy2 ratio. Spots that are more abundant in control or knockdown samples are indicated on the schematic gels by the white rectangles. Comparison across gels was carried out by inter-gel matching using Cy2 images (pooled internal standard) and DeCyder ™ BVA software module (GE healthcare). Following gel-to-gel matching of spots, univariate statistical analysis (Student's t-test) of the log standardized abundance changes between groups was performed using DeCyder BVA software module. PLS-DA multivariate statistical analysis, combined with an iterative threshold process to identify which protein spots had the greatest contribution to the model (Karp et al., 2005; Karp et al., 2004), was used to identify spots that significantly changed. Combination of both statistical analyses, together with manual inspection, led to identification of 81 spots that had changed upon PTB+nPTB siRNA treatment. Spots were picked and analyzed by LC-MS/MS sequencing. MASCOT reports allowed identification of proteins within the spots. If peptides identified by MASCOT were specific to an alternative splicing event, primers were designed and RT-PCR analysis was carried out to validate the event. In cases where peptide coverage did not discriminate between spliced isoforms, the UCSC Genome Browser was used to identify candidate PTB-regulated AS events in the gene of interest.

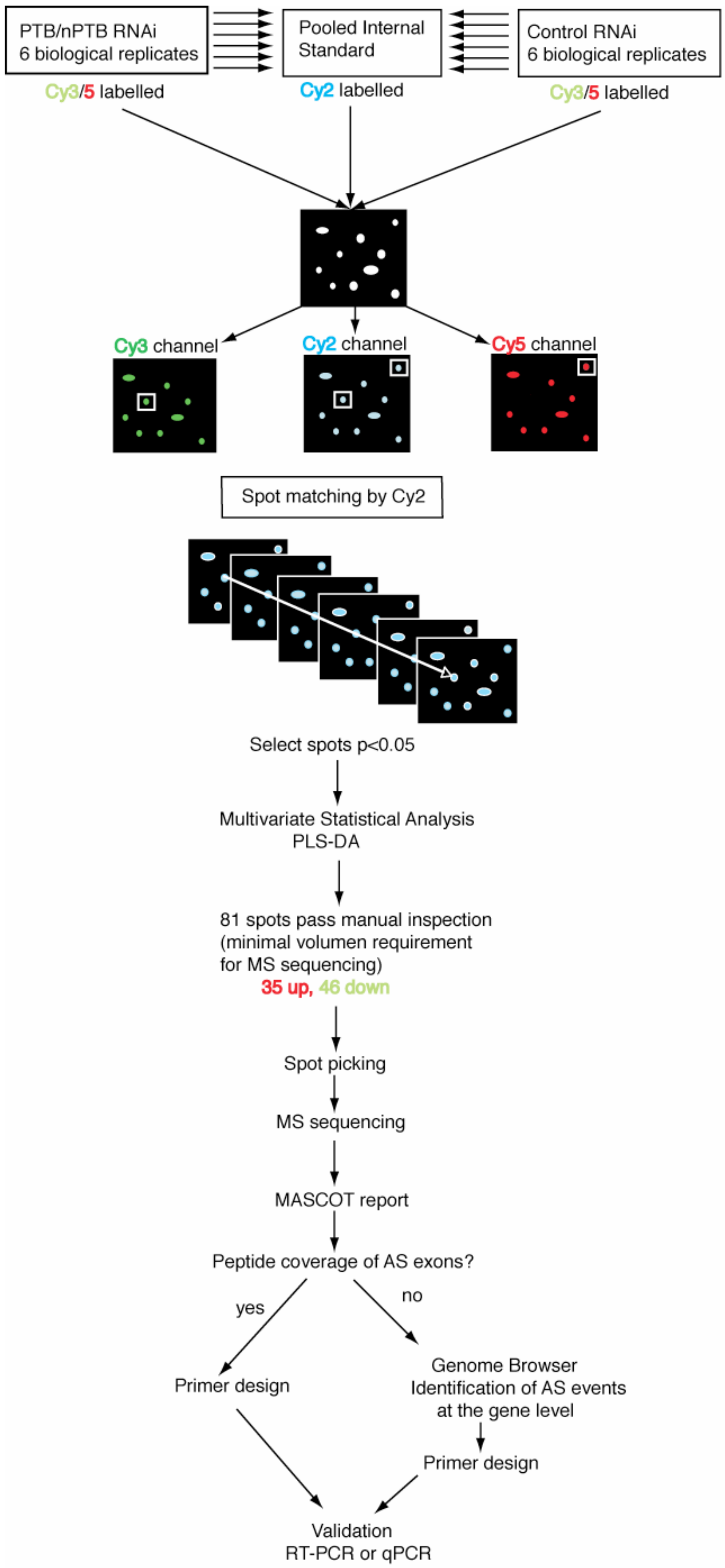
PTB/nPTB RNAi
6 biological replicates
Pooled Internal
Standard
Control RNAi
6 biological replicates
Cy3/5 labelled
Cy2 labelled
Cy3/5 labelled
Cy3 channel
Cy2 channel
Cy5 channel
Spot matching by Cy2
Select spots p<0.05
Multivariate Statistical Analysis
PLS-DA
81 spots pass manual inspection
(minimal volumen requirement
for MS sequencing)
35 up, 46 down
Spot picking
MS sequencing
MASCOT report
Peptide coverage of AS exons?
yes
no
Primer design
Genome Browser
Identification of AS events
at the gene level
Primer design
Validation
RT-PCR or qPCR
